# Pedaling Performance Changing of Elite Cyclists Is Mainly Determined by the Fatigue of Hamstring and Vastus Muscles during Repeated Sprint Cycling Exercise

**DOI:** 10.1155/2020/7294820

**Published:** 2020-01-03

**Authors:** Lejun Wang, Qineng Shao, Guoqiang Ma, Mingxin Gong, Wenxin Niu, Jun Qiu

**Affiliations:** ^1^Sport and Health Research Center, Physical Education Department, Tongji University, Shanghai, China; ^2^Physical Education and Sports Science Institute of Shanghai, Shanghai 200030, China; ^3^Shanghai Yangzhi Rehabilitation Hospital, Tongji University School of Medicine, Shanghai 201619, China

## Abstract

Repeated sprint cycling is an effective training method in promoting athletic performance of cyclists, which may induce severe fatigue of lower limb muscles. However, the relationship between the fatigue of each lower limb muscles and the changing of exercise performance remains unclear. In this study, ten cyclist volunteers performed a series of 6-second sprints with 24-s recovery for five times. Power, cadence, and EMG mean frequency (MNF) of each lower limb muscle group for every 2-second epoch, as well as the grey relational grade between exercise performance and MNF of each lower limb muscle group during the whole process were calculated. It has been found that MNF of Rectus femoris (RF), Vastus (VAS), Gastrocnemius (GAS), and the hamstring muscle group (HAM) showed significant negative correlation with the increase in both sprint number and intrasprint duration time, while the grey relational grade of HAM and VAS was higher than that of other muscles. The results demonstrated that the exercise performance of both power and cadence were most closing related to the fatigue degree of HAM and VAS during repeated sprint cycling exercise.

## 1. Introduction

Repeated sprint cycling exercise is an effective training method in promoting athletes performance and has been widely used in sport training for cyclists [1, 2]. During sprint pedaling, lower limb muscles perform rapid high-intensity contractions and may induce severe fatigue in less than 5 seconds [3]. During repeated sprint cycling, with the increase of movement time and repeated number, the fatigue of most of lower limb muscles enhance progressively, resulting in significant decrease in exercise performance [4]. When performing sprint exercise, the performance of the exercise is mainly determined by the ability of muscles to produce and maintain maximal levels of power [5]. Exploring the relationship between the fatigue of each lower limb muscle and the changing of exercise performance may provide insights in further understanding the movement and designing sport training programs.

In previous research studies, activities and coordination of each lower limb muscle during fatigue-free and prolonged (i.e., 30 s) sprint cycling has been investigated extensively [5–8]. As referred to the fatigue evaluation of lower limb muscle fatigue, previous research studies have mainly focused on the fatigue assessment of only 1 to 2 muscles [3, 9, 10]. As different muscles may play different functional roles during pedaling exercise, the fatigue development process of each lower limb muscle may differ [5, 6]. For example, muscle activity of the quadriceps has been found to decrease by ∼8%, whereas gastrocnemius (GAS) has shown a decrease of up to 15% during 30-s sprint cycling, indicating an unbalanced development of fatigue for the two muscle groups [11, 12]. However, most of the lower limb muscles' fatigue degree has not been quantified simultaneously during sprint cycling. In particular, no studies have investigated the relationship between the fatigue degree of each lower limb muscle and the exercise performance during repeated sprint cycling.

Surface EMG (sEMG) has been widely used in muscle fatigue evaluation [13–15]. It has been demonstrated that surface EMG indices may show steady changing tendency during isometric and dynamic fatiguing contraction of both sustained and intermittent exercise [14]. In particular, Wang et al. [9] have found that mean frequency (MNF) derived from wavelet packet transformation of EMG is superior to other indices in muscle fatigue evaluation induced by sprint cycling exercise, which may provide advices for surface EMG-based muscle fatigue evaluation during repeated sprint cycling exercise. On the other hand, grey relational analysis proposed by Deng [16] is a good method to determine the influence and contribution of the independent variables to the observed results [17, 18]. It has been suggested to be suitable for solving problems with complicated interrelationships between multiple factors and variables, which may provide insights to quantify the influence of each lower limb muscle fatigue to the exercise performance.

The aim of this study is to determine the influence of each lower limb muscle (or muscle group) fatigue to the exercise performance during repeated sprint cycling exercise. Fatigue of each muscle is estimated by MNF calculated based on wavelet packet transformation of sEMG. Grey relational grade between MNF of each muscle and exercise performance (power and cadence) were calculated and compared. We hypothesize that the fatigue of different lower limb muscles may influence unequally to the exercise performance, which may be demonstrated by significant different grey relational grade between exercise performance indices and MNF of different muscles.

## 2. Materials and Methods

### 2.1. Participants

Seven male and three female highly trained competitive elite sprint cyclist volunteers (age 21.50 ± 4.67 years, height 175.00 ± 8.25 cm, and weight 75.40 ± 10.91 kg) participated in this study. The cyclists had received 6 days per week and 8 hours per day cycling training for more than 7 years and competed in Chinese national track cycling events. None of the cyclists has received ergogenic aids or PED's. Participants had been asked to refrain from strenuous physical activity 24 hours before the experiment. They were screened using a questionnaire to ensure that they had not suffered lower body injury or other health issues that affected performance. The experimental design of the study was approved by the Ethics Committee of Tongji University and was explained to the participants before they gave their written informed consent. The study was performed during the racing season of the cyclists.

### 2.2. Experimental Protocol

The experiment was conducted in a laboratory with the indoor temperature of about 24°C and comprised a warm-up exercise and repeated sprint cycling exercise. All the exercises were performed on an air-braked ergometer (Wattbike Pro; Wattbike Ltd., Nottingham, United Kingdom) that allows the resistance to be set between 1 and 10 levels. The Wattbike measures the forces applied to the chain over a load cell and angular velocity of the crank twice per revolution to calculate the power output at a rate of 100 Hz. After the test, power and cadence results recorded by using a Wattbike ergometer can be exported for every 1 second.

The warm-up exercise consisted of a 5-min cycling exercise with the air resistance on the ergometer set at level 3 and the cadence at 90 rpm followed by a rest of 10 min mixed with complete rest and gentle stretching exercises. Based on previous tests with the subjects, the air resistance on the Wattbike ergometer was set to the level at which the subject may produce the maximum power output during sprint cycling. According to this criterion, the air resistance on the ergometer was set to level 8∼10 for seven male cyclists and level 6∼8 for three female cyclists. According to the calibration report, level 6 and 10 of air resistance on the Wattbike ergometer result in power outputs of 45 and 55 W at a cadence of 40 rpm and 785 and 1045 W at a cadence of 130 rpm.

**Figure 1:**
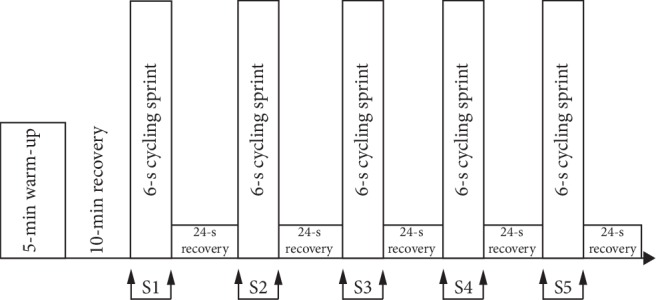
Experimental setup. The testing protocol is composed of 5 × 6-s sprints with 24-s recovery time between them. Subjects performed a submaximal cycling exercise at 60 rpm with the air resistance on the ergometer set to level 1, before and after repeated sprints.

The repeated sprint cycling exercise is composed of 5 × 6-second sprints with 24-s recovery time between them. For each sprint, a rolling start was adopted in which participants start to pedal at a low force level and the test load was imposed only when the cadence reached to 60 rpm. During each sprint cycling, participants performed all-out cycling sprint with seated position. They were encouraged to produce the highest possible power output for 6 seconds and were verbally encouraged throughout the trials. Subjects performed a submaximal cycling exercise at 60 rpm with the air resistance on the ergometer set to level 1 during a 24-second recovery time. An overview of the testing procedures is provided in Figure 1.

During the test, the position of the participants on the ergometer was adjusted accordingly to the setup of the cyclists' own bike, and the crank length was 170 mm in the whole experiment. During pedalling exercise, the feet of the subjects were fixed to the pedal via two straps during the pedalling exercise. Surface EMG signals and Wattbike data were synchronized by a trigger which can start the EMG and Wattbike data sampling software simultaneously.

### 2.3. EMG Measurement

Surface EMG were recorded from eight muscles of the right lower limb (rectus femoris (RF), vastus lateralis (VL), vastus medialis (VM), biceps femoris (BF), semitendinosus (ST), tibialis anterior (TA), gastrocnemius lateralis (GAS), and soleus (SOL)). Muscles from the same functional group were averaged to produce EMG_VAS_ (EMG_VL_ + EMG_VM_) and EMG_HAM_ (EMG_BF_ + EMG_ST_) [5].

Surface EMG were recorded with bipolar Ag/AgCl electrodes of an ME 6000 P8 Surface EMG acquisition instrument (Mega Electronics System, Finland). Electrodes were placed over the belly of muscles with center-to-center electrode distance setting to 2 cm. The skin was shaved and cleaned with alcohol wipes before the electrodes were fixed. Medical adhesive tape and plastic casts were applied to fix the electrodes. The EMG signals were amplified, band-pass filtered (3–1000 Hz), digitized (1000 samples/s), and acquired by the MegaWin system (Mega Electronics System, Finland).

### 2.4. EMG, Power, and Cadence Data Processing

EMG signals were band-pass filtered at 5–500 Hz offline using a 4^th^ order zero-phase-shift Butterworth filter and were divided into every 2-second epochs for each 6-second sprint cycling exercise. Accordingly, power and cadence were averaged for every 2-second epochs for each subject during the repeated sprint cycling.

For each epoch, EMG MNF based on wavelet packet transformation were calculated. Wavelet packet transformation was employed to analyze sEMG, and a wavelet that was a member of the Daubechies family (order 6) was implemented in this analysis [19]. On this basis, MNF was calculated based on the following formula:

(1) $$\begin{array}{c} \text{MNF}=\frac{\int_{0}^{\infty }\omega P\left(t,\omega \right)\text{d}\omega }{\int_{0}^{\infty }P\left(t,\omega \right)\text{d}\omega }, \end{array}$$

where *P*(*t*, *ω*) represents the power spectrum of EMG signals based on wavelet packet transformation.

EMG MNF as well as power and cadence of each subject calculated for 2-second epochs were normalized as

(2) $$\begin{array}{c} X_{\text{Normalized}}=\frac{X_{i}-X_{\min}}{X_{\max}-X_{\min}}, \end{array}$$

where *X*_Normalized_ represents the normalized value of raw data *X*_*i*_. *X*_max_ and *X*_min_ represent the maximum and minimum value of series *X*.

### 2.5. Bland–Altman Analysis

The results of power and MNF of each muscle were input into the MedCalc software for Bland–Altman analysis, and the deviation diagram was drawn. The Bland–Altman deviation diagram is a two-dimensional Cartesian coordinate system, the *X*-axis of the abscissa represents the average value of the two results, and the *Y*-axis of the ordinate represents the difference and the average value between two results. Also, it is recommended that 95% of the data points should lie within ±1.96 SD of the mean difference [20].

### 2.6. Grey Relational Grade Calculation

In the grey relational grade calculation, EMG MNF of six muscle groups were selected as inspected sequences while power or cadence was chosen as the standard sequence. Each sequence data was normalized by dividing the average value of the sequence. Then, the grey relational coefficient was calculated using Deng's grey relational grade formula [21]:

(3) $$\begin{array}{c} \text{corr}\left(x_{0}\left(k\right),x_{i}\left(k\right)\right)=\frac{\Delta \min+p\Delta \max}{\Delta_{0i}\left(k\right)+p\Delta \max}, \end{array}$$

where

1. *i* = 1, 2, 3,…, *m*, *k* = 1, 2, 3,…, *n*.
2. *x*_0_: standard sequence. *X*_*i*_: inspected sequence.
3. Δ_0*i*_ = ||*x*_0_(*k*)−*x*_*i*_(*k*)||: the difference between *x*_0_ and *x*_i_.
4. Δ_min._=∀_*i*_^min.min.^∀ *k*‖*x*_0_(*k*) − *x*_*i*_(*k*)‖, Δ_max._ = ∀_*i*_^max.max.^∀ *k*||*x*_0_(*k*)−*x*_*i*_(*k*)||.
5. *p*: distinguishing coefficient and *p* ∈ [0,1]. In this study, we took *p*=0.5 according to previous research [9].

When the grey relational coefficient is calculated, the mean value of the grey relational coefficient is taken as the grey relational grade:

(4) $$\begin{array}{c} \text{CORR}\left(x_{0},x_{i}\right)=\frac{1}{n}\overset{n}{\underset{k=1}{\sum }}\text{corr}\left(x_{0}\left(k\right),x_{i}\left(k\right)\right). \end{array}$$

The grey relational grade of CORR in this study ranged from 0 to 1. A larger value of CORR indicates more proximity of changing trends between EMG index and power or cadence and thus a significant influence on exercise performance.

Data processing was performed using MATLAB R2016a software (Mathworks, USA).

### 2.7. Statistical Analysis

The statistical analysis was performed using SPSS 13.0 for windows (SPSS, Inc., Chicago, IL, USA). Normality was tested using the Kolmogorov–Smirnov test. A repeated-measures analysis of variance (within factors: sprint number (1, 2,…, 5) and intrasprint duration time (2, 4, and 6 second)) was used to compare the difference of dependent variables (power, cadence, and EMG MPF). A repeated-measures analysis of variance was also used to compare the difference of grey relational grade (within factors: muscles (RF, VAS, HAM, TA, GAS and SOL) and exercise performance (power vs. cadence)). A *t*-test with Bonferroni correction was implemented when appropriate. Spearman rank correlation analysis was used to observe the correlation between dependent variables (power, cadence, and EMG MNF) and sprint number, as well as intrasprint duration time. Paired *t*-test was employed to test the difference of dependent variables before and after recovery time. All significant thresholds were fixed at *α* = 0.05.

## 3. Results

**Figure 2:**
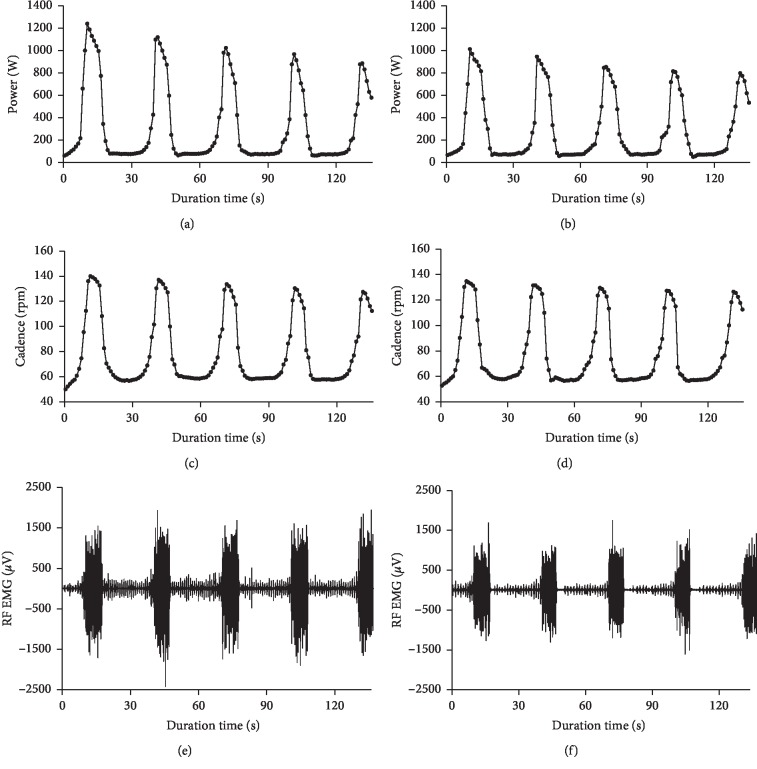
Power output (a) and (b), cadence (c) and (d), and raw EMG signals of rectus femoris (e) and (f) for two representative subjects during the repeated sprint cycling exercise.

Examples of power output, cadence, and raw EMG signals of rectus femoris for two representative subjects during the repeated sprint cycling exercise are shown in Figure 2. It can be observed from the figure that power output, cadence, and EMG amplitude showed high values during the sprint cycling exercise while the values decreased to substantially low levels during the recovery time.

**Figure 3:**
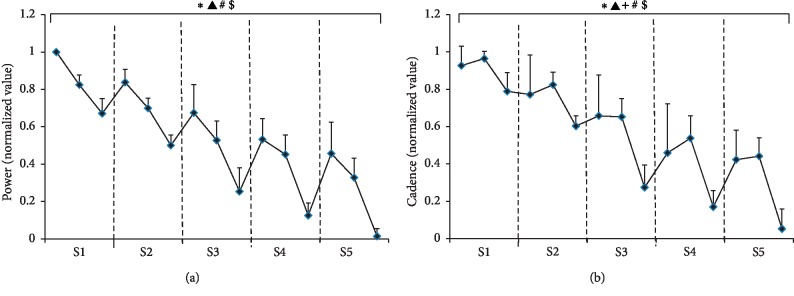
Average power output (a) and cadence (b) of all subjects calculated for every 2-second duration time during five repeated sprint cycling exercise. The results for each bout were separated by dashed lines. ^*∗*^ means a significant main effect of sprint number, ▲ means a significant main effect of intrasprint duration time, and +means a significant interaction influence between sprint number and intersprint duration time on power (a) and cadence (b), while # and $ indicate significant correlation between exercise performance (power and cadence) and sprint number or intersprint duration time.

Figure 3 shows the average power output (a) and cadence (b) of all subjects calculated for every 2 seconds during five repeated sprint cycling exercise. Power and cadence decreased progressively with the increase in both sprint number and intersprint duration time. Significant negative correlations were revealed between exercise performance (power and cadence) and sprint number, as well as intersprint duration time (sprint number, power: *r* = −0.546, *P* ≤ 0.001; duration time, power: *r* = −0.407, *P* ≤ 0.001; sprint number, cadence: *r* = −0.681, *P* ≤ 0.001; and duration time, cadence: *r* = −0.327, *P* ≤ 0.001). A repeated-measures analysis of variance demonstrated that both sprint number and intersprint duration time had significant influence on the power output and cadence (sprint number, power: *F* = 157.565, *P* ≤ 0.001; duration time, power: *F* = 208.420, *P* ≤ 0.001; sprint number, cadence: *F* = 128.232, *P* ≤ 0.001; and duration time, cadence: *F* = 57.609, *P* ≤ 0.001). A significant interaction influence was found between sprint number and intersprint duration time on cadence (*F* = 3.373, *P* < 0.01). Besides, power and cadence showed significant increase after recovery time (power: *P* ≤ 0.001; cadence: *P* ≤ 0.001).

**Figure 4:**
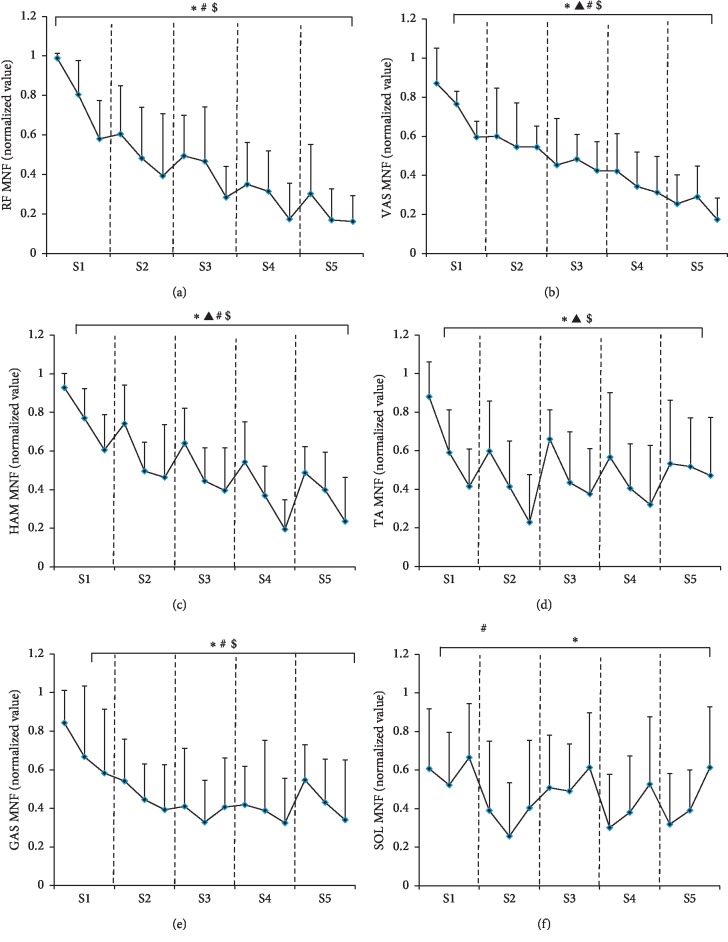
EMG MNF of each muscle calculated for every 2 seconds during five repeated sprint cycling exercise. The results for each bout were separated by dashed lines. ^*∗*^ means a significant main effect of sprint number, while ▲ means a significant main effect of intrasprint duration time on EMG MNF, while # and $ indicate significant negative correlation between EMG MNF and sprint number or intersprint duration time.

Figure 4 shows the EMG MNF of each muscle calculated for every 2 seconds during five repeated sprint cycling exercise. Spearman cross-correlation analysis revealed that MNF of RF, VAS, HAM, and GAS decreased significantly with the increase of sprint number (RF: *r* = −0.610, *P* ≤ 0.001; VAS: *r* = −0.720, *P* ≤ 0.001; HAM: *r* = −0.509, *P* ≤ 0.001; TA: *r* = −0.121, *P* > 0.05; GAS: *r* = −0.284, *P* ≤ 0.001; and SOL: *r* = −0.127, *P* > 0.05). On the other hand, MNF of RF, VAS, HAM, TA, and GAS showed significant decrease in tendency with the increase in intrasprint duration time (RF: *r* = −0.163, *P* ≤ 0.05; VAS: *r* = −0.166, *P* ≤ 0.05; HAM: *r* = −0.492, *P* ≤ 0.001; TA: *r* = −0.435, *P* ≤ 0.001; GAS: *r* = −0.235, *P* ≤ 0.01; and SOL: *r* = 0.122, *P* > 0.05). A repeated-measures analysis of variance demonstrated that sprint number had a significant influence on the MNF of each muscle (RF: *F* = 24.925, *P* ≤ 0.001; VAS: *F* = 47.709, *P* ≤ 0.001; HAM: *F* = 20.116, *P* ≤ 0.001; TA: *F* = 2.821, *P* ≤ 0.05; GAS: *F* = 9.443, *P* ≤ 0.001; and SOL: *F* = 2.742, *P* ≤ 0.05), while intrasprint duration time had significant influence on the MNF of VAS, HAM, and TA (RF: *F* = 1.834, *P* > 0.05; VAS: *F* = 4.649, *P* ≤ 0.05; HAM: *F* = 34.366, *P* ≤ 0.001; TA: *F* = 18.521, *P* ≤ 0.001; GAS: *F* = 2.648, *P* > 0.05; and SOL: *F* = 3.372, *P* > 0.05). No significant interaction influence was found between sprint number and intersprint duration time on each muscle (RF: *F* = 0.799, *P* > 0.05; VAS: *F* = 1.733, *P* > 0.05; HAM: *F* = 0.816, *P* > 0.05; TA: *F* = 1.318, *P* > 0.05; GAS: *F* = 0.523, *P* > 0.05; and SOL: *F* = 1.056, *P* > 0.05). Besides, only MNF of HAM and TA showed significant increase after recovery time, while MNF of SOL showed significant decrease after recovery time (RF: *P* > 0.05, VAS: *P* > 0.05, HAM: *P* ≤ 0.001, TA: *P* ≤ 0.001, GAS: *P* > 0.05, and SOL: *P* ≤ 0.05).

**Figure 5:**
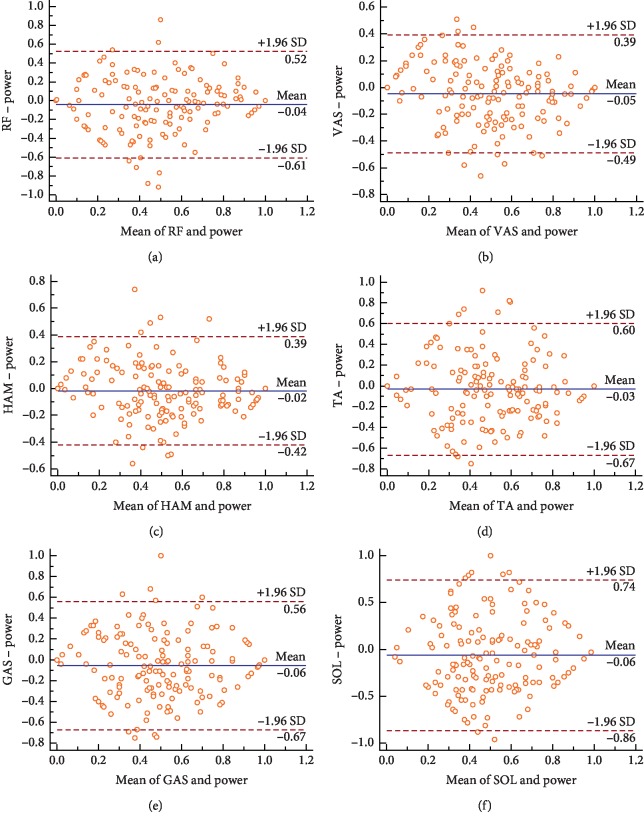
Bland–Altman plots comparing the RF (a), VAS (b), HAM (c), TA (d), GAS (e), and SOL (f) of EMG MNF vs. power. The dotted lines represent 95% limits of agreement.

Figure 5 shows the results of Bland–Altman analysis between power and EMG MNF of each muscle. The results showed that MNF of RF, VAS, TA, GAS, SOL, and power results within the 95% consistency limit (RF: 96.0%, VAS: 95.3%, HAM: 95.3%, TA: 95.3%, GAS: 95.3%, and SOL: 95.3%) were over 95%, which met the consistency requirements.

Table 1 shows the grey relational grade of EMG indices and pedalling performance. Statistics revealed that muscle factor had a significant influence on the grey relational grade value (*F* = 25.754, *P* ≤ 0.001). No significant main effect of performance indices (*F* = 0.233, *P* > 0.05) as well as interaction influence between EMG and performance indices was found (*F* = 0.956, *P* > 0.05). Multicomparison results revealed that the grey relational grade of HAM and VAS was higher than that of other muscles (*P* < 0.05). Grey relational grade of RF was higher than TA and SOL, while SOL showed the minimum value among all the muscles.

## 4. Discussion

The aim of this study is to explore the relationship between the exercise performance and lower limb muscle fatigue. It has been found that MNF of RF, VAS, HAM, and GAS all showed significant negative correlation with the increase of both sprint number and intrasprint duration time, while the grey relational grade of HAM and VAS were higher than other muscles. As far as we know, it is the first time to compare the influence of each lower limb muscle fatigue to the exercise performance during repeated sprint cycling.

During sprint cycling, lower limb muscles performed vigorous high force contraction. It has been suggested that most of the lower limb muscles were recruited at high activation level during sprint cycling exercise [6, 7, 10]. In previous research, peak muscle activities of VAS, RF, and GAS during sprint cycling has been found to be nearly 100% of the maximal value in isometric contractions [8]. The high activation of VAS, RF, and GAS muscle may be close related to the significant fatigue development of the three muscle groups with the increase in both sprint number and intrasprint duration time in the current research. Besides, as the compositions of type II muscle fibres for RF and HAM are as high as 60% and 55%, which are much high than most lower limb muscles and may be apt to induce muscle fatigue during sprint cycling [22, 23]. On the other hand, the fatigue degree of TA and SOL was not as severe as other muscles, which may be explained by the lower activation level as well as high composition of type I muscle fibres [22, 23]. As MNF of RF, VAS, HAM, and GAS showed the same changing tendency with power and cadence during sprint cycling exercise, the close relationship between the fatigue of RF, VAS, GAS, and HAM and the changing of exercise performance can be expected.

During submaximal cycling exercise, previous research has revealed that monoarticular knee extensors (VAS) and plantar flexors (GAS and SOL) were the most activated lower limb muscles [8], the workload of which was similar to the recovery pedalling exercise adopted in this study. Moreover, RF has been found to be more susceptible to fatigue development than other lower limb muscles during submaximal as well as sprint cycling exercise [5, 22, 23]. Therefore, it can be inferred that RF, VAS, GAS, and SOL may not tend to recover from fatigue during recovery time. In this study, only MNF of HAM and TA showed significant increase after recovery time, indicating that only the fatigue of HAM and TA recovered significantly, the results of which was consistent with the conclusion mentioned above. The results may illustrate the significant role of HAM and TA in the power and cadence increase after recovery time.

In this study, the grey relational grades of HAM and VAS were higher than other muscles, indicating that the fatigue of HAM and VAS were most closely related to the decrease in exercise performance during the repeated sprint cycling. The key role of VAS for the total power contribution during maximal sprint cycling has been revealed by previous research using muscle functional magnetic resonance imaging [24]. Therefore, it can be inferred that the significant influence of VAS for the changing of exercise performance can be related to the significant fatigue development as well as the main power producer role of the VAS muscle.

However, the predominant role of HAM for the power produced has rarely been suggested during continuous sprint cycling exercise in previous research studies [5]. In the current research, HAM was the only muscle the MNF of which not only significant decreased with both sprint number and intrasprint duration time but significantly increased as a result of recovery adjustment. The decrease in MNF indicated a significant fatigue development, while the increase in MNF may demonstrate a fatigue recovery of HAM after recovery time. Therefore, the significant role of HAM to the influence of exercise performance may be related to the significant fatigue development in cycling sprint as well as the fatigue recovery in recovery time.

However, it is necessary to consider the limitations of gender effect on the results as the number of male and female subjects is distinct in the current study. Aiming at this problem, firstly, previous research has revealed no significant difference of power decrease rate between male and female subjects during repeated sprint cycling exercise with the similar protocol of the current study [25]. Second, the changing rate of surface EMG indices including motor unit action potential conduction velocity and fractal dimension showed no significant gender distinction during the fatiguing task till exhaustion [26]. Lastly, we have compared the power output, cadence and MNF for each muscle and have demonstrated that the above indices of male and female subjects showed very similar changing tendency and no significant difference was found between male and female subjects. Therefore, it can be concluded that the gender factor may have no significant influence on the results of this study. Another limitation that should be addressed is that a number of lower limb muscles, such as gluteus maximus (GMax) and medial gastrocnemius (GAS_M_), have not been included in the current research. In previous research studies, the muscles of GMax, ST, BF, VM, RF, VL, GAS_M_, GAS_L_, SOL, and TA have been deemed to be the most important muscles contributing to pedalling exercise [6]. It has been suggested that the functional role of each muscle depends on how many joints the muscles traverse [6]. So, it can be inferred that GAS_M_ and GAS_L_ may play approximately the same functional role, and the limitation of the absence of GAS_M_ can be ignored in the current study. However, GMax is the only monoarticular muscle of the hip extension in the abovementioned ten lower limb muscles, indicating that the functional role of GMax cannot be replaced by other muscles and the absence of GMax indeed is a nonnegligible limitation. Despite the limitations mentioned above, the current research still provides insights for the understanding of the relationship between the fatigue of lower limb muscles and exercise performance of repeated sprint cycling.

## 5. Conclusions

In conclusion, RF, VAS, HAM, and GAS fatigued progressively with the increase in both sprint number and intrasprint duration time, while only the fatigue of HAM and TA recovered significantly after recovery time. The fatigue degree of HAM and VAS were most close related to the changing of exercise performance during the repeated sprint cycling. The key role of VAS is related to the significant fatigue development as well as the role of main power producer during cycling sprint, while the influence of HAM may be related to the significant fatigue development in sprint cycling as well as the fatigue recovery in recovery time. The findings may provide insights for the coaches and cyclists to further understand the movement and design training program.

## Figures and Tables

**Table 1:** Grey relational grade between pedalling performance and EMG MNF of each lower limb muscle.

	RF	VAS	HAM	TA	GAS	SOL
Power	0.68 ± 0.02^*∗*^	0.74 ± 0.07	0.75 ± 0.06	0.67 ± 0.06	0.67 ± 0.05	0.61 ± 0.05
Cadence	0.68 ± 0.05	0.74 ± 0.04	0.77 ± 0.05	0.63 ± 0.04	0.65 ± 0.04	0.61 ± 0.05

Note: ^*∗*^a significant main effect of muscle factor on the grey relational grade value.

## Data Availability

The data used to support the findings of this study are available from the corresponding author upon request.
